# Nanolayered CoCrFeNi/Graphene Composites with High Strength and Crack Resistance

**DOI:** 10.3390/nano12122113

**Published:** 2022-06-20

**Authors:** Xiaobin Feng, Ke Cao, Xiege Huang, Guodong Li, Yang Lu

**Affiliations:** 1 Hubei Key Laboratory of Theory and Application of Advanced Materials Mechanics, Wuhan University of Technology, Wuhan 430070, China; 13264702577@163.com; 2 School of Mechano-Electronic Engineering, Xidian University, Xi’an 710071, China; caoke@xidian.edu.cn; 3 CityU-Xidian Joint Laboratory of Micro/Nano-Manufacturing, Shenzhen 518057, China; 4 State Key Laboratory of Advanced Technology for Materials Synthesis and Processing, Wuhan University of Technology, Wuhan 430070, China; 5 Department of Mechanical Engineering, City University of Hong Kong, Hong Kong 999077, China

**Keywords:** high-entropy alloy, monolayer graphene, nanolayered composites, strength, crack resistance

## Abstract

Emerging high-entropy alloy (HEA) films achieve high strength but generally show ineludible brittle fractures, strongly restricting their micro/nano-mechanical and functional applications. Nanolayered (NL) CoCrFeNi/graphene composites are elaborately fabricated via magnetron sputtering and the transfer process. It is uncovered that NL CoCrFeNi/graphene composite pillars exhibit a simultaneous ultra-high strength of 4.73 GPa and considerable compressive plasticity of over 20%. Detailed electron microscope observations and simulations reveal that the monolayer graphene interface can effectively block the crack propagation and stimulate dislocations to accommodate further deformation. Our findings open avenues for the fabrication of high-performance, HEA-based composites, thereby addressing the challenges and unmet needs in flexible electronics and mechanical metamaterials.

## 1. Introduction

In the last two decades, high-entropy alloys (HEAs) have shown important mechanical properties for potential engineering applications due to their extensively tunable compositions and microstructures [1]. The complex hierarchical structure from the atomic to the macroscopic level endows HEAs with excellent mechanical properties, including high specific strength, yield strength, and fracture toughness at a wide range of temperatures [2,3]. Based on these intrinsic features, magnetron co-sputtering deposition is regarded as one of the promising high-throughput techniques to provide great scope for performance optimization [4]. In this case, extensive attempts have been made to fabricate HEAs in the form of a film, which exhibits high strength [5], thermal stability [6], and corrosion resistance [7] designed for advanced coating applications, such as diffusion barriers [8] and metallic nano-/micro-lattices [9]. Especially, researchers found that nanocrystalline HEA films exhibit fundamentally different mechanical properties compared with corresponding bulk forms. For example, the hardness of a nanocrystalline CoCrFeNiAl_0.3_ HEA film was measured to be 7.66 GPa: much stronger than its bulk form [10]. Another example is that 250 nm thick nanocrystalline NbMoTaW HEA film showed a peak hardness of 5.9 GPa: ~5–6 times higher than that of bulk NbMoTaW HEA [11,12]. The significant improvement in strength promotes nano-grained HEA films at a low cost in engineering applications.

It is normally expected that bulk HEAs with coarse grains generally demonstrate decent ductility but insufficient strength, i.e., far below their ideal strength. However, with regards to polycrystalline HEAs, grain refinement strengthens materials via the classical Hall–Petch relation while significantly depressing ductility. As grain sizes fall into the nanoscale, limited deformability becomes the Achilles’ heel of strong, nanocrystalline HEA films [13]. Meanwhile, microstructures of nanocrystalline materials are generally unstable: stress- and thermal-driven grain coarsening occurs even at low temperatures, thereby strongly deteriorating their mechanical properties [14]. Therefore, knowing how to maintain strength without sacrificing ductility is highly desirable for both scientific interests and practical applications. A few promising methods have been adopted to stabilize nanostructures and enhance the mechanical properties of metals, such as alloying [15,16] and nitridation [17]. Still, most of them are illustrated as playing limited roles in improving comprehensive mechanical behaviors. In this case, synthesis of nanolayered films with controllable, individual layer thickness is achieved via magnetron sputtering alternate deposition [18]. These nanolaminates have drawn worldwide attention due to their introduction of unique interfaces and building of constitutional layers [19]. CoCrFeNi is one of the well-studied FCC HEA systems and is generally shortlisted as a model HEA [7,10,20]. For example, previous studies on Cu/CoCrFeNi nanolayered micropillars fabricated by focused ion beam (FIB) uncovered that the strength and deformation mechanisms show a strong layer thickness dependence [20]. At larger layer thickness, a homogenous-like deformation was shown in a fashion that was pillar diameter independent [20]. This finding paves a new way to achieve the important mechanical properties of HEAs.

In parallel, it is well realized that graphene is one of the promising, low-dimensional materials with superior strength, modulus, and thermal conductivity, making it an attractive proposition for flexible electronics applications [21,22]. Recently, freestanding monolayer graphene exhibited an engineering tensile strength of ~50–60 GPa, a Young’s modulus up to ~1 TPa, and an elastic strain approach of ~6%, indicating near-ideal mechanical performance [23]: much higher than that of multilayer graphene nanosheets [24]. In addition to the intrinsic, superb mechanical properties of monolayer graphene, the heterogeneous metal–graphene interface renders much higher, critical resolved shear stress for dislocation cross-slip/nucleation in the vicinity of the grain boundaries, leading to an obvious increase in strength in Cu/graphene and Al/graphene composites [25,26]. Benefiting from the graphene interface, Cu/graphene and Ni/graphene composites show superior strength as much as 31% over the theoretical strengths [27]. However, most of the reported graphene transfer approaches contain the Cu foil etching process, which is complicated to process and is proven to introduce extrinsic metallic ions [25,26,27]. Meanwhile, it is a great challenge to obtain simultaneous high strength and good ductility by manipulating compositional and microstructural features. Therefore, the feasible and tunable fabrication of high-performance NL metal/graphene composites, especially in HEA/graphene systems is highly desirable for both scientific interests and practical applications.

Motivated by the above issues, in this work, we combined two promising model materials, CoCrFeNi HEA film and monolayer graphene, to carefully prepare the CoCrFeNi/graphene nanolayered composites. First, the modified fabrication method is systematically presented. The mechanical properties of CoCrFeNi/graphene nanolayered micropillars were investigated. Furthermore, a combined experimental and computational methodology was used to yield the underlying deformation and fracture mechanisms.

## 2. Materials and Methods

### 2.1. Material Preparation

**Figure 1:**
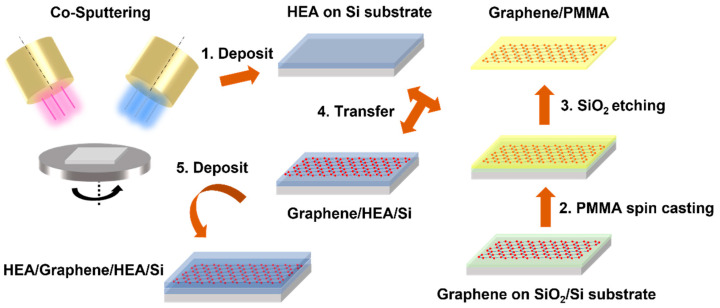
Schematic figure of the fabrication of NL HEA/graphene composites.

As shown in Figure 1, there are overall five steps to fabricating a NL CoCrFeNi/graphene composite. First, the magnetron co-sputtering technique was employed to correspondingly deposit 625 nm thick CoCrFeNi HEA film on the (100)-oriented Si wafers at room temperature. The film thickness was regulated by working time when other sputtering parameters were kept constant. The working distance between the target and Si wafer was ~90 mm. The sputter power was set at 30 W. The argon flow (99.99%) was controlled and fixed at 20 sccm (standard cubic centimeter per minute), while the working pressure was set as 0.3 Pa. The rotation speed was 30 rpm (rotation per minute) for the homogeneous deposition. Second, chemical vapor deposited monolayer graphene on SiO_2_/Si substrate (6Carbon Technology, Shenzhen, China) and was spin-coated with poly-(methyl methacrylate) (PMMA) as the protective layer, followed by heating at 180 °C for one min—curing. Third, the SiO_2_/Si template was etched away by KOH solution (1 mol/L) at 90 °C. Subsequently, the freestanding graphene supported by PMMA was released into distilled water three times to remove the KOH solute. Fourth, the graphene with PMMA coating was transferred to the as-prepared 625 nm thick CoCrFeNi HEA film on Si substrate and then left in a dry (40% relative humidity) cabinet overnight at 25 °C so that the graphene attached firmly to the HEA film. Finally, the PMMA layer was removed after immersing in acetone, and the ~1250 nm thick CoCrFeNi/graphene composite was fabricated, followed by, again, deposition with the same parameters as in the first step.

### 2.2. Material Characterization and Mechanical Testing

The morphology and thickness of CoCrFeNi HEA film were characterized by the field emission scanning electron microscope (SEM, FEI, Columbus, OH, USA) equipped with energy-dispersive X-ray (EDX, Oxford, UK). X-ray diffraction (XRD, Rigaku SmartLab, Tokyo, Japan) was used to detect the crystal structure. Transmission electron microscopy (TEM, JEOL 2100F, 80 kV, and 200 kV, Tokyo, Japan) was used to determine the microstructure, and the TEM foil was fabricated by focused ion beam (FIB, FEI, Columbus, OH, USA) milling. The NL CoCrFeNi/graphene micropillars with 850 nm diameter were fabricated using FIB. The FIB milling was conducted with a gallium ion beam with the parameters of initial voltage between 30 kV and 15 kV and current from 1 nA to 1.5 pA to reduce gallium contamination. To determine the mechanical properties of NL composites, uniaxial compression testing was conducted on the NL CoCrFeNi/graphene micropillars with 850 nm diameter by nanoindentation (TI950, Hysitron, Billerica, MA, USA) equipped with a commercial flat punch tip (5 μm) at the quasi-static strain rate at room temperature. The initial contact area and height of the micropillar were used to obtain the engineering stress–strain curves. The reproducibility of curves was evaluated by testing three times.

### 2.3. Simulation

Molecular dynamics (MD) simulations were performed to illustrate the deformation and fracture behaviors, especially in the vicinity of the CoCrFeNi/graphene interface, using LAMMPS software. Specifically, embedding atomic potential was used to display the interatomic interaction in the HEA [28]. The Tersoff potential was employed in the C–C interaction [29]. The interactions among metallic Co, Cr, Fe, and Ni atoms and C atoms also needed to be considered for atomic scale simulations by developing the Lennard–Jones potential [30]. Here, we built the CoCrFeNi/graphene atomic configuration considering orientations X = $\left[11\bar{2}\right]$_CoCrFeNi_||[100]_Gr_, Y = [111]_CoCrFeNi_||[010]_Gr_, and Z = $\left[1\bar{1}0\right]$_CoCrFeNi_||[001]_Gr_; note that Gr is graphene. The dimensions of the initial model were L_x_ = 201.128 Å, L_y_ = 206.114 Å, and L_z_ = 201.95 Å. Before loading, the whole system was equilibrated at 1 K for 100 ps via the Nosé–Hoover isothermal–isobaric (NPT) ensemble, resulting in L_x_ = 199.479 Å, L_y_ = 205.718 Å, and L_z_ = 200.292 Å (a = 3.538 Å). For loading experiments, the micropillars were aligned along the Y-direction, while periodic boundary conditions were used in three axes. Simulations were performed at a constant strain rate (ε) of 109/s using the Nosé–Hoover canonical ensemble (NVT).

## 3. Results and Discussion

**Figure 2:**
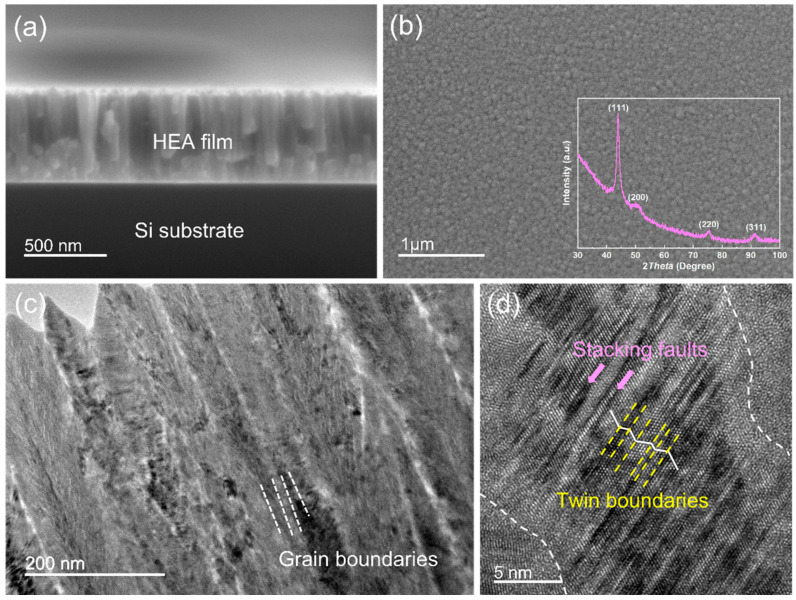
Characterization of CoCrFeNi HEA film. (**a**) The SEM cross-sectional observation of CoCrFeNi HEA film; (**b**) the SEM image of the HEA film surface inserted with the XRD patterns; (**c**) a representative TEM cross-sectional observation of CoCrFeNi HEA film; (**d**) the corresponding HRTEM image.

Field emission scanning electron microscope (SEM, FEI) observations were used to understand the growth and morphology of CoCrFeNi HEA films. Columnar grains with high aspect ratios were observed on the cross-sectional HEA film with a film thickness of 625 nm, as shown in Figure 2a. In Figure 2b, one can see that the SEM observation on the surface of the HEA films revealed homogeneous spherical grains. To detect the crystal orientation, we performed the X-ray diffraction (XRD, Rigaku SmartLab, Tokyo, Japan) on the as-fabricated HEA film; see the insert in Figure 2b. The CoCrFeNi films exhibited strong (111)-oriented and weak (200)-, (220)-, and (311)-oriented textures, manifesting the face-centered cubic (FCC) structure. Figure 2c shows typical cross-sectional transmission electron microscopy (TEM, JEOL 2100F, 200 kV, Tokyo, Japan) observations of HEA film. It was found that columnar nanocrystalline grains were embedded with a handful of planar defects. Figure 2d shows the corresponding high-resolution TEM (HRTEM) image, indicating the existence of dense planner defects, i.e., stacking faults (SFs) and nanotwins (NTs).

**Figure 3:**
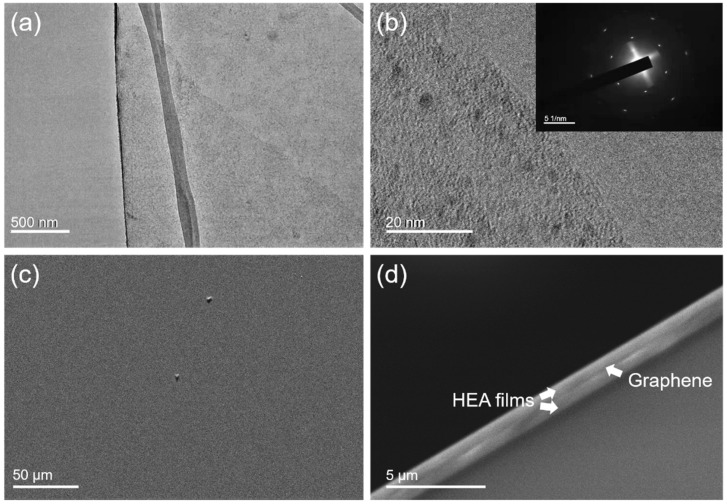
Characterization of the monolayer graphene and its assembling. TEM observation of (**a**) the freestanding graphene before transfer and (**b**) the edge of the suspended graphene; SEM observation of (**c**) graphene on the HEA surface and (**d**) cross-sectional NL HEA/graphene composite.

The morphology and structure of the monolayer graphene were studied by TEM. Figure 3a exhibits the low, magnified view of the freestanding graphene before the transfer process. It shows a typical, wrinkled surface without evident holes or cracks, indicating superior quality. The edges of the suspended graphene were studied via HRTEM operating at 80 kV. As shown in Figure 3b, there was merely one area of dark contrast in the edge area, and the inserted selected area diffraction pattern (SADP) in Figure 3b demonstrates the single hexagonal diffraction pattern, and, therefore, the single crystalline and monolayer nature was confirmed. After graphene was transferred to the HEA/substrate, followed by removing the PMMA, SEM observation was conducted on the graphene/HEA/substrate sample, as shown in Figure 3c. The surface of the graphene was smooth with a few residual PMMAs or contaminants above and below the graphene, showing the successful transfer process. The cross-sectional SEM observation on the as-fabricated HEA/graphene/HEA composites is shown in Figure 3d, which depicts a sharp interface between two HEA layers, indicating a solid connection between the two HEA films by the monolayer graphene.

**Figure 4:**
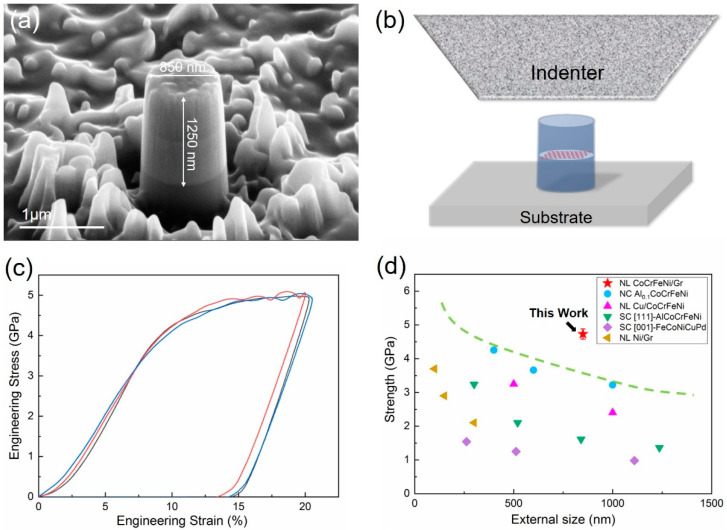
Illustration of (**a**) as-fabricated NL HEA/graphene micropillar; (**b**) the uniaxial micro-compression mechanical testing; (**c**) uniaxial compressive stress–strain curves of NL HEA/graphene micropillars; (**d**) the strength vs. the external size of the NL HEA/graphene micropillar compared with other related materials, including nanocrystalline (NC) Al_0.1_CoCrFeNi HEA film pillar, NL CoCrFeNi/Cu micropillar, single crystalline (SC) [111]-orientated AlCoCrFeNi HEA pillar, SC [001]-orientated FeCoNiCuPd HEA pillar, and NL Ni/graphene pillar.

The NL CoCrFeNi/graphene micropillars with 850 nm diameter were fabricated by FIB, as can be seen in Figure 4a. To determine the mechanical properties of NL composites, uniaxial compression testing was conducted on the NL CoCrFeNi/graphene micropillars, as shown in the schematic Figure 4b. Figure 4c exhibits the typical compressive stress–strain curves of the NL HEA/graphene micropillar. The stress–strain curves demonstrate a steady plastic flow without evident strain burst even when the strain was up to 20%. Note that the flow stress measured at a strain of 5% of the NL HEA/graphene micropillar was chosen as the strength, following the prevalent approach [31,32]. The strength of the NL HEA/graphene micropillar and other, related small-sized materials as a function of external size is plotted in Figure 4d. Compared with other related materials at small scale, including a nanocrystalline (NC) Al_0.1_CoCrFeNi HEA film pillar [33], NL CoCrFeNi/Cu micropillar [20], single crystalline (SC) [111]-orientated AlCoCrFeNi HEA pillar [5], SC [001]-orientated FeCoNiCuPd HEA pillar [34], and NL Ni/graphene pillar [27], the presenting NL HEA/graphene micropillar exhibited unprecedented compressive strength exceeding 4.7 GPa.

**Figure 5:**
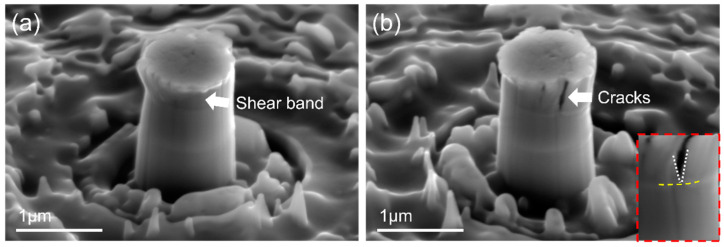
Postmortem SEM (**a**) front view; (**b**) side view of deformed NL HEA/graphene micropillar.

To reveal the underlying deformation and fracture behaviors, postmortem SEM observations were performed on the NL HEA/graphene micropillar after micro-compression testing. A representative SEM image of the NL micropillar displayed a localized shear deformation at the upper part of the HEA that yielded at 20% compressive engineering strain, as shown in Figure 5a. The side view of Figure 5b shows a few large cracks propagating along the compressive axis. It is worth mentioning that the propagation of a large crack was arrested at the graphene interface, which is evident in the enlarged image in Figure 5b. The cracking and shearing events are captured well with minor stress fluctuations in the stress–strain curve in Figure 4c.

For the HEA matrix, the superior strength first was attributed to a high density of interior barriers (grain boundaries and nanotwins) for dislocation movement. Since there is relatively small grain size in CoCrFeNi HEA films, most emitted dislocations cannot accumulate within grains. Thus, dislocations are more likely to be emitted from the surface or interface and glide within the narrow columns to sustain the plastic deformation [35]. Ultra-thin nanotwins are suggested to soften materials as a dislocation-nucleation-controlled softening mechanism [36]. However, when mobile dislocations encounter numerous inclined nanotwins, we hypothesize that the dislocations are inclined to cut nanotwins into segments, which are additional spinning points that block the following dislocation motion, imparting the high strength. As for the fracture behaviors, i.e., the early crack generated in HEA film, the crux of the problem lies in the high aspect ratio of HEA grains, as experimentally proved by the SEM and TEM observations. The CoCrFeNi HEA films populated with columnar grains suffer from easier buckling events in terms of Euler’s criterion [37,38]. Particularly, a larger aspect ratio sustains stronger cohesive strength of grain boundaries to block buckling. In such a condition, columnar grains tend to buckle and prompt the entire structure to lose its load-bearing limit, and, consequently, the fracture comes about the individual grain boundary cracking. Heavy nanotwins embedded in the nano-sized grains of the HEAs are suggested to accommodate the dislocation motion and cater to the further plastic deformation and delay the crack propagation [39].

**Figure 6:**
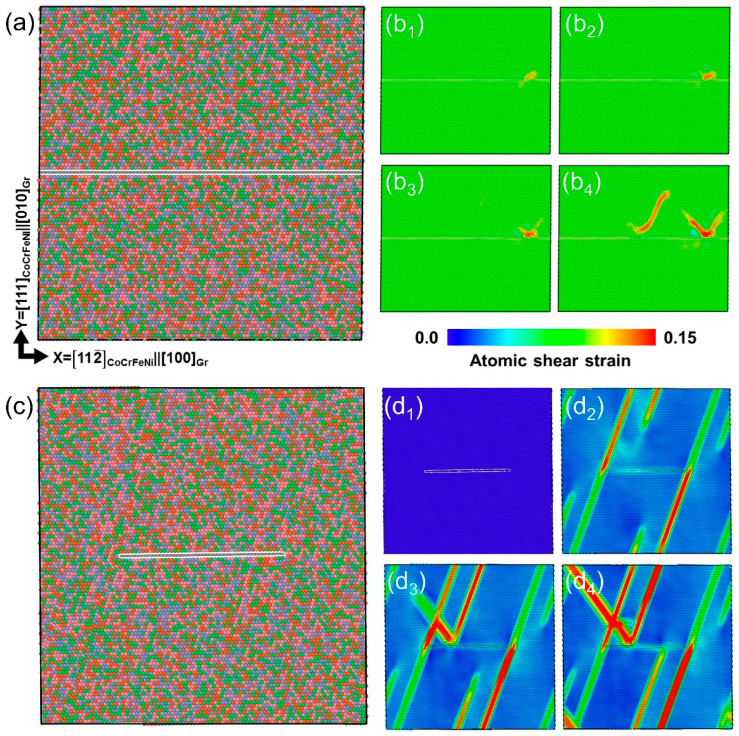
(**a**) The CoCrFeNi/graphene/CoCrFeNi atomic configuration; (**b1**–**b4**) the corresponding atomic shear strain maps at the compressive strain of 16.0, 16.1, 16.2, and 16.4%; (**c**) the CoCrFeNi/intermittent graphene/CoCrFeNi atomic configuration; (**d1**–**d4**) the corresponding atomic shear strain maps at the compressive strain of 0, 5.4, 5.6, and 6.0%.

More importantly, the experimental observation on crack features indicated that the monolayer graphene interface can strongly hinder and arrest crack propagation. To better understand the effect of the graphene interface on the HEA matrix, MD simulation was employed on an interfacial model system of X = $\left[11\bar{2}\right]$_CoCrFeNi_||[100]_Gr_, Y = [111]_CoCrFeNi_||[010]_Gr_, and Z = $\left[1\bar{1}0\right]$_CoCrFeNi_||[001]_Gr_, as shown in Figure 6a. It is suggested in Figure 6b that the dislocation was first emitted from the inner HEA and moved towards the monolayer graphene interface as the atomic shear was applied at a 16.0% strain and was subsequently hindered by the monolayer graphene. As the strain was up to 16.2%, a parallel dislocation was initiated and eventually blocked by the monolayer graphene. No obvious step was found in graphene after obstructing several dislocation activities, which was a result of a ~1 TPa measured modulus in monolayer graphene [23], restricting dislocation activities within the upper part of the HEA/graphene composites and, therefore, effectively strengthening the HEA matrix. It is worth mentioning that mirror-like dislocation emission and motion were found after encountering the graphene barrier. Analogous to stimulated emission in laser theory, which has been theoretically and experimentally proved in Cu and Ti alloys [40,41], the dislocation slip can be promoted to the next layer to sustain the deformation and decrease the stress concentration near the interface. In this case, such stimulated slip endows dislocation storage near the interface and in the upper part of HEA inner grains during continuous deformation.

**Figure 7:**
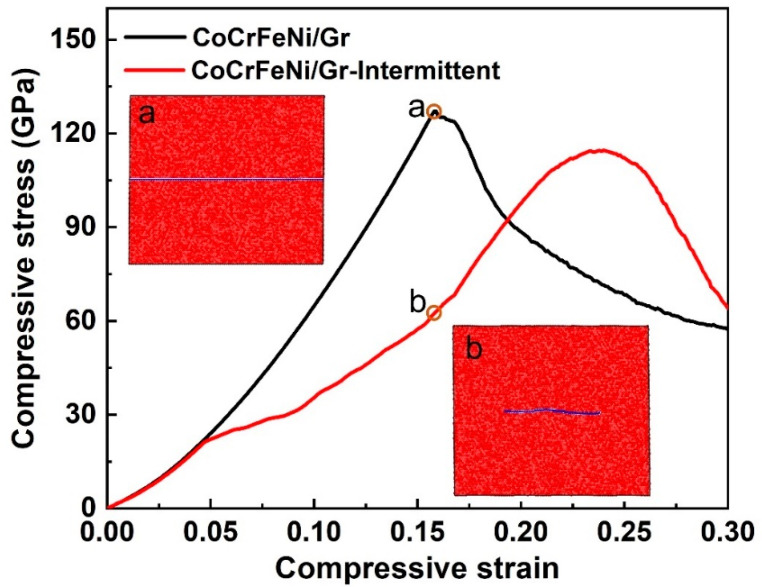
The calculated compressive stress–strain curves of NL CoCrFeNi/Gr composites with continuous graphene and intermittent graphene inserted with the corresponding configurations of graphene at 15.9% compressive strain.

Note that sputtering and transfer might induce damages, such as holes, vacancies, delamination, etc. In this case, the atomic configuration of the CoCrFeNi/intermittent graphene/CoCrFeNi was also considered, as shown in Figure 6c. Figure 6(d1–d4) contains atomic shear strain maps that exhibit the corresponding compressive strain at 0, 5.4, 5.6, and 6.0%. Different from the continuous graphene layer, in this model system, some dislocations were emitted and reached the other end of the HEA layer without any interference (Figure 6(d2)), while others were impeded by the intermittent graphene and deflected as the strain increased further (Figure 6(d3,d4)). Therefore, the intermittent graphene acted as a weak interface to block dislocation motions to decrease strength but increase the dislocation pathway to some extent, resulting in a lower ultimate compressive strength and prolonged compressive strain compared to that of the continuous interface, as shown in Figure 7. Interestingly, inserted corresponding configurations of continuous graphene (a) and intermittent graphene (b) at a 15.9 % compressive strain revealed that intermittent monolayer graphene tends to bend to accommodate higher stress concentration during deformation, suggesting that damages in graphene may not deteriorate the mechanical performance of the NL CoCrFeNi/Gr composites.

## 4. Conclusions

To sum up, we systematically introduced the fabrication, mechanical properties, and deformation behaviors of nanolayered CoCrFeNi/graphene composites. The main findings are as follows:

(1) Coupled with HEA sputtering and high-quality monolayer graphene transfer techniques, nanolayered CoCrFeNi/graphene composites can be delicately fabricated;
(2) The presented CoCrFeNi/graphene nanolayered composite pillars showed a superior high strength of 4.73 GPa exceeding 20% compressive strain;
(3) By postmortem microscope observations and molecular dynamics simulations, the simultaneous high strength and deformability can be interpreted by nanotwins in the HEA matrix and the monolayer graphene interface as a result of confining the dislocation pathway and stimulating dislocations emission and storage.

Our preliminary findings not only provide insights into the mechanical properties of CoCrFeNi/graphene nanolayered composites but also offer some clues to the tunable design and feasible fabrication of high-performance, metallic-based composites.

## Data Availability

Not applicable.
